# Biotransformation of α-Acetylbutyrolactone in *Rhodotorula* Strains

**DOI:** 10.3390/ijms19072106

**Published:** 2018-07-20

**Authors:** Wanda Mączka, Katarzyna Wińska, Małgorzata Grabarczyk, Barbara Żarowska

**Affiliations:** 1 Department of Chemistry, Wrocław University of Environmental and Life Sciences, Norwida 25, 50-375 Wrocław, Poland; katarzyna.winska@upwr.edu.pl (K.W.); magrab@onet.pl (M.G.); 2 Department of Biotechnology and Food Microbiology, Wrocław University of Environmental and Life Sciences, Chełmońskiego 37/41, 51-630 Wrocław, Poland; barbara.zarowska@upwr.edu.pl

**Keywords:** yeast, biotransformation, lactone, DES, glycerol

## Abstract

Due to its structural similarity, the α’-1′-hydroxyethyl-γ-butyrolactone obtained by reduction of (±)-α-acetyl-γ-butyrolactone may have a similar function in the body to γ-butyrolactone (GBL). In the work presented, biotransformation of α-acetyl-γ-butyrolactone by three *Rhodotorula* strains was performed obtaining enantiomerically enriched alcohol. The process was carried out in growing and resting cultures. We studied how both media composition and organic solvent volume affected stereoselectivity and effectiveness of biotransformation. After 2 h, the enantiomerically pure (3*R*, 1′*S*)-α’-1′-hydroxyethyl-γ-butyrolactone was obtained using the *R. marina* AM77 strain in YPG (Yeast extract-Peptone-Glucose) medium enriched with 5% glycerol. To our best knowledge there is no previous information in the literature about the (±)-α-acetyl-γ-butyrolactone biotransformation performed in medium with addition of organic and deep eutectic solvents.

## 1. Introduction

*Rhodotorula* is included in the order *Sporidiobolales*, class *Microbotryomycetes*, and phylum Basidiomycota in the Fungi kingdom [1]. Species of this genus are ubiquitous saprophytic yeasts that can be acquired from many environmental sources including air, soil, grass, lakes, oceans, food, and human skin [1,2]. *Rhodotorula*’*s* biomass can also be a valuable addition to animal feed because these strains synthetize carotenoids: β-carotene, torulene, and torularhodin (a typical feature of this species is red colour of cells) [3,4]. The lipid content in *R. glutinis* biomass can reach up to 60% [4].

In our study of effective reduction of (±)-α-acetyl-γ-butyrolactone **1**, we chose *Rhodotorula* strains, because of their known ability to commonly reduce low-molecular-weight ketones—derivatives of acetophenone [5,6,7,8,9]—and ketoesters [10]. 

The product of α-acetylbutyrolactone reduction are enantiomers of α’-1′-hydroxyethyl-γ-butyrolactone **2**, which could be potential central nervous system (CNS) ligands. This compound is a structural analog of γ-butyrolactone (GLB) which exerts several inhibitory actions over CNS through interaction with specific neuronal high-affinity receptors [11]. Additionally, because it is a bifunctional compound (lactone and alcohol), it could be a useful building block in asymmetric synthesis [12]. In biological tests it is essential to use compounds in their pure enantiomer forms, especially when synthesizing pharmaceuticals, where a high enantiomeric excess is required to prevent undesired side effects by the second enantiomer [13].

Contrarily, biotransformation could be ineffective, when substrates are toxic or have low solubility in water. Overcoming these limitations is accomplished by creating an organic-aqueous biphasic system. The aqueous phase constitutes a buffer or medium along with biocatalyst. The second phase is a water immiscible phase; therefore, both the substrate and the product strongly prefer this phase. During the reaction this phase acts as a substrate reservoir, and on the other hand as an in situ extractant for the product [14]. Presently, deep eutectic solvent (DES) is a new designer solvent, which is a mixture of two or more components. It forms a eutectic with the melting point lower than each individual component separately [15,16,17]. The most popular halide salt among all DES is choline chloride (ChCl), which is similar to B-class vitamins and it is a biodegradable and nontoxic salt [17]. As hydrogen bond donor (HBD) glycerol is most often used in DES. This compound is also the conventional solvent extensively used in food and pharmaceutical industries [18].

DES based on ChCl and glycerol is hydrophilic but its polarity does not influence catalytic activity. Viscosity has the ability to change enzyme activity in reaction systems by changing the mass-transfer limitations. DES are inexpensive, nontoxic, biodegradable, and do not require further purification [16].

In this paper, we describe biotransformation of (±)-α-acetyl-γ-butyrolactone **1** by means of *Rhodotorula* strains in both grown and resting cells culture in presence of different solvents and DES.

## 2. Results and Discussion

**Figure 1:**
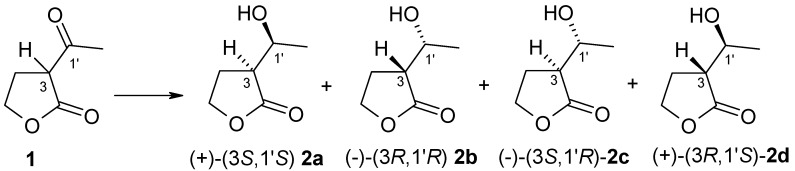
Structures of substrate **1** and stereoisomers of product **2**.

The first step of our investigation was the chemical reduction of (±)-α-acetyl-γ-butyrolactone **1** to obtain product standard, which was used in the chiral column (GC) analysis. The reaction was carried out according to the procedure worked out by Teixeira et al. [12]. As result of this chemical reaction we received *anti*-to-*syn* isomers of α’-1′-hydroxyethyl-γ-butyrolactone **2** in a ratio of 1:7. We used strain of *Yarrowia lipolytica* P26A to obtain (3*R*,1′*R*)-α’-1′-hydroxyethyl-γ-butyrolactone **2b** [19] (Supplementary Materials). We compared the values of the specific rotation of obtained enantiomers of **2** with literature data [19,20]. To assign GC signals to the corresponding alcohol isomers, both chemically obtained **2** and the results of biotransformation of this substrate were used. As a result of the studies, it was possible to determine retention time of individual alcohol stereoisomers **2a**–**2d** (Figure 1).

Thus, we started a screening procedure using three yeast strains: *Rhodotorula glutinis* AM242, *Rhodotorula marina* AM77 and *Rhodotorula rubra* C9, which were grown in different media. YPG medium (Medium A) has a rich composition, as yeast extract is present alongside peptone as a source of nitrogen. We wanted to investigate if the change in nutrient composition would affect the transformation. We used the most universal Sabouraud medium (Medium B) only consisting of glucose and peptone, and lastly MCM (Mushroom Complete Medium) medium (Medium C) with a reduced amount of peptone and yeast supplemented with mineral salts. The samples were collected after 1, 2, 3, 4 and 5 h (Table 1, Table 2 and Table 3).

In the case of α-acetylbutyrolactone **1** transformation by the *R. glutinis* AM242 strain, the highest degree of conversion was observed in medium A (YPG) (Table 1). The substrate was transformed for five days, thereafter the amount of unreacted substrate was less than 35%. In addition, the presence of 1% yeast extract in the medium facilitated the formation of the *anti* diastereoisomer. In the other media, a constant dominance of the *syn* diastereoisomer was observed. In addition, when using B and C media, the biotransformation process was much slower, because after five days, about 70% of the unreacted substrate remained.

The *R. marina* AM77 strain was found to be a much better biocatalyst in the transformation of **1** than *R. glutinis* AM242 because the complete substrate conversion occurred in just 3 h both in Medium A and B (Table 2). The presence of peptone as a source of nitrogen in concentration of at least 1% (S) seems to be significant for substrate conversion. 

In the case of **1** biotransformation by the *R. rubra* C9 strain, the highest substrate conversion and transformation diastereoselectivity were observed using YPG medium (Medium A) (Table 3). This strain proved to be a much better biocatalyst than *R. glutinis* AM242 and comparable to *R. marina* AM77 in terms of reactivity of the substrate, considering that complete substrate conversion was achieved after three hours of transformation. The use of B or C media increased the biotransformation time to four hours.

The subsequent stage of our research was to investigate the stereoselectivity in biotransformation. The tests were performed in the optimal medium identified for each strain in the initial studies. Transformations were finished after three hours in the cases of *R. rubra* C9 and *R. marina* AM77, and after five hours for *R. glutinis* AM242 (Table 4).

The main product of the transformation of α-acetylbutyrolactone **1** using all strains was the **2d** stereoisomer, with the exception of *R. glutinis* AM242 strain where in biotransformation the formation of all four stereoisomers was observed. 

The presence of enol (Table 1, Table 2 and Table 3) as well as the low diastereoselectivity and enantioselectivity of the transformation 1 by *R. glutinis* AM242 (Table 4) is in accordance with mechanism described by Ribeiro [11]. The formation of all four stereoisomers is due to the reduction of both the enol and the carbonyl group of **1**.

In the consecutive experiment, biotransformation of substrate **1** in resting culture was investigated. For this purpose, the harvested cells was suspended in phosphate buffer at pH = 7. Biotransformations were carried out for two days with sampling at 3, 5, 8, 24 and 48 h. The sampling was finished after complete conversion of substrate **1** (Table 5).

In this case, the time required to complete conversion of substrate **1** was prolonged in comparison to the results presented previously. *R. glutinis* AM242 required 48 h for complete conversion, and strains of *R. marina* AM77 and *R. rubra* C9 needed 8 h and 5 h, respectively. The highest enantioselectivity was observed for the *R. marina* AM77 strain, as only products **2b** and **2d** were obtained. The strain of *R. rubra* C9 was also capable of producing an enantiomerically pure **2d** enantiomer and a mixture of **2a** and **2b** isomers with the latter’s superiority.

Because the medium contains a high concentration of glucose, which facilitates the regeneration of enzyme cofactors, we have decided to check whether the addition of sugar will favour biotransformation of **1**. For this purpose, in next experiment, the 1% glucose was added to harvested cells of *Rhodotorula* strains suspended in phosphate buffer at pH = 7 (Table 6).

Glucose supplementation did not significantly affect transformations using the resting cell culture of *R. glutinis* AM242. More differences were examined in the other two strains. However, *R. marina* AM77 and *R. rubra* C9 strains formed **2a** isomer in addition to the **2b** isomer, which reduced the enantiomeric excess of stereoisomer *syn*. Furthermore, *R. rubra* C9 strain exhibited a significant increase in the percentage of pure enantiomerically **2d** stereoisomer.

In the last phase of our study we examined the effect of solvent addition on the substrate conversion. The studies were carried out using strain of *R. marina* AM77, while taking into account both types and amounts of solvent applied. The biotransformation was finished after 2 h (Table 7).

In the aforementioned experiments (Table 4, Table 5 and Table 6), the formation of all four stereoisomers of **2** in different proportions was observed. Each organic solvent added to the medium had individually different although significant effects on diastereo- and enantioselectivity of biotransformation. In almost all cases (except for the presence of 20% glycerol), the formation of the stereoisomer **2d** alone was observed. Presence of solvent in transformation mixture may have inhibited enzymes responsible for reduction of the double bond in enol. Hence the **2d** stereoisomer was produced by stereoselective reduction of the carbonyl group. Unfortunately, the addition of solvents had a negative effect on the substrate’s reactivity. Total conversion of **1** occurred only in one transformation, associated with adding 5% glycerol to the medium.

In case of carbonyl reductase, 2-propanol can be used as co-substrate for regeneration of NAD+ or NADP+ and increases substrate solubility [21]. Supplementation with isopropanol (5%) improved the stereoselectivity of the transformation. A considerably larger amount of isopropanol (20%) completely inhibits enzymes responsible for this transformation. In turn, ethanol proved to be an interesting alternative to glucose as co-substrate to regenerate cofactors. However, it is toxic to the cells at higher concentration [22].

Scrutinizing the results for full conversion of substrate **1** to one (3*R*, 1′*S*)-enantiomer **2d** after two hours, the transformation by *R. marina* AM77 in presence of 5% glycerol was chosen for preparative scale biotransformation. Next, optical rotation was determined after purification of **2d** by means of column chromatography. The obtained value of optical rotation (${\left[a\right]}_{20}^{D}\;$= +18.4 (c = 0.7; CHCl_3_), was compared with literature data to confirm configuration of the biotransformation product [20].

Meanwhile biotransformation **1** performed by the *R. marina* AM77 strain in medium coupled with glycerol had a favourable effect on the process. Next, we carried out transformation in a phosphate buffer pH = 7 with DES. The deep eutectic solvent was a mixture of choline chloride and glycerol in a ratio of 1:2 or a mixture of chloride choline, glycerol and glucose in a ratio of 1:2:1. The samples were collected after 5 h, 1 day, 2 days, 4 days and 7 days (Table 8). 

The use of DES in biotransformations by means of resting cells negatively affected the process. The addition of DES resulted in significant prolonged time of all biotransformations and reduced stereoselectivity. More frequently, the isomers **2a**, **2b** and **2d** were formed. Formation of pure isomers **2b** and **2d** in combination with complete conversion of substrate **1** was observed only in the presence of ChCl:Gly at 10% and ChCl:Gly:Glc at 25%.

## 3. Materials and Methods 

### 3.1. Analysis

Progress of all biotransformations and purity of isolated products were checked by TLC on silica gel-coated aluminium plates (DC-Alufolien Kieselgel 60 F254, Merck, Darmstadt, Germany) and also by GC analysis performed on a CP03380 instrument (Varian, Agilent Technologies, Santa Clara, CA, USA) using an DB-1 column (dimethylpolysiloxane, Agilent, 30 m × 0.25 mm × 0.25 µm). Temperatures during GC analysis were as follows: injector 250 °C, detector (FID) 300 °C, column temperature: 75 °C (hold 3 min), 75–80 °C (rate 2 °C/min), 80–150 °C (rate 17 °C/min), 150–300 (rate 40 °C/min), 300 °C (hold 1 min). The enantiomeric excess of the products obtained during biotransformation were determined by GC analysis using chiral column Gamma DEX^TM^ 325 (30 m × 0.25 mm × 0.25 µm, Supelco, Bellefonte, PA, USA) under the following conditions: injector 150 °C, detector (FID) 250 °C, column temperature: 80 °C (hold 20 min), 80–107 °C (rate 1 °C/min), 107–200 °C (rate 30 °C/min), 200 °C (hold 1 min). All products were purified using preparative column chromatography on silica gel (Kieselgel 60, 230–400 mesh). NMR spectra were recorded with a Bruker Avance DRX-500 spectrometer in CD_3_OD solution. Optical rotations were measured on P-2000 polarimeter (Jasco, Easton, PA, USA). 

### 3.2. Synthesis of Standards

The chemical reduction of α-acetylbutyrolactone **1** was carried out according to the procedure worked out by Teixeira et al. [12]. α-Acetylbutyrolactone **1** (1 g, 7.8 mmol) in 30 mL of methanol was stirred for 30 min at room temperature in the presence of CaCl_2_ (1.73 g, 15.6 mmol). Next, the reaction mixture was cooled to 0 °C and NaBH_4_ was slowly added. When reaction was complete, crude product was extracted by diethyl ether (3 × 15 mL) and dried over MgSO_4_. After evaporation 0.68 g of α’-1′-hydroxyethyl-γ-butyrolactone **2** was obtained.

^1^H NMR (500 MHz, CD_3_OD, Figure S1): 1.25 (d, *J* = 7.0 Hz, 3H, CH_3_-2′B), 1.26 (d, *J* = 6.4 Hz, 3H, CH_3_-2′A), 1.22–1.24 (dq, *J* = 12.8 and 8.5 Hz, 1H, one of CH_2_-2A), 2.22–2.30 (m, 1H, one of CH_2_-2B), 2.32–2.39 (m, 2H, one of CH_2_-2A and one of CH_2_-2B), 2.65 (ddd, *J* = 9.4, 9.4 and 3.3 Hz, 1H, H-3B), 2.74 (ddd, *J* = 9.4, 8.8 and 4.7 Hz, 1H, H-3A), 4.05 (dq, *J* = 6.4 and 4.8 Hz, 1H, 1′A), 4.22–4.27 (m, 2H, CH_2_-1A), 4.33–4.38 (m, 3H, CH_2_-1B and 1′B).

### 3.3. Microorganisms

The yeast strains of *Rhodotorula glutinis* AM242, *Rhodotorula marina* AM77, *Rhodotorula rubra* C9 were used in this research. The microorganisms were obtained from collection of the Department of Chemistry or the Department of Biotechnology and Food Microbiology at Wroclaw University of Environmental and Life Sciences (Wrocław, Poland). Stock cultures of these strains were maintained on a gelified medium (glucose 20 g/L, peptone 20 g/L, yeast extract 10 g/L and agar 20 g/L) and were stored at 4 °C.

### 3.4. Cultivation Media

Medium A (YPG) was composed of: (g/L) glucose (20), peptone (20), yeast extract (10).

Medium B was composed of: (g/L) glucose (30), peptone (10).

Medium C (MCM) was composed of: (g/L) glucose (20), peptone (2.0), yeast extract (2.0), KH_2_PO_4_ (0.49), K_2_HPO_4_ (1.0), MgSO_4_ × 7H_2_O (0.5).

### 3.5. Screening Procedure

The microorganisms were cultivated at 25 °C in 300 mL Erlenmeyer flasks containing 50 mL of the appropriate medium. After 4 days of growth, 50 µL of substrate (0.060 g, 7.8 mmol) was added to the shaken cultures. The transformation was continued for maximum 4 days. The medium (20 mL) contained unreacted substrate, product and mycelium were extracted with ethyl acetate (20 mL). The organic fraction were dried over anhydrous magnesium sulphate and the solvent was evaporated *in vacuo* and analysed by GC (DB-1 column).

### 3.6. Preparative Biotransformation

The culture of *Rhodotorula marina* AM77 (50 mL, 12.0 g/L) was centrifuged at 5000 rpm for 3 min. The culture was re-suspended in two flasks with 100 mL of sterile medium with addition of 5% glycerol. In next step, the substrate (100 mg) was added. After two hours, the medium, which contained product and mycelium was extracted three times with ethyl acetate (100 mL). The organic fraction were dried over anhydrous magnesium sulphate, the solvent was evaporated *in vacuo* and analysed by GC (chiral column). Product of biotransformation was purified by means of column chromatography (Kieselgel 60, 230–400 mesh; hexane:diethyl ether 1:1, 67 mg, 67%). Next, the optical rotation was determined. The obtained value of optical rotation (${\left[a\right]}_{20}^{D}$ = +18.4 (c = 0.7; CHCl_3_) was compared with literature data [20]. Therefore, the biotransformation product is (3*R*, 1′*S*)-α’-1′-hydroxyethyl-γ-butyrolactone (**2d**).

### 3.7. Preparation of Resting Cell Suspension

The cultivated cells were harvested by centrifugation (5000 rpm, 3 min) at 4 °C and washed three times with distilled water. Resting cells of microorganism were re-suspended in 40 mL of 0.1 M phosphate buffer (KH_2_PO_4_ + Na_2_HPO_4_, pH = 7.0) to make a resting cell suspension (6.0 g/L).

### 3.8. Biotransformation in the Presence of Organic Solvents

The culture of microorganism (20 mL, 12.0 g/L) was centrifuged at 5000 rpm for 3 min. The culture was re-suspended in 50 mL of sterile medium with addition of appropriate organic solvent (ethanol, isopropanol, glycerol, hexane). In the next step the substrate was added. After suitable time, the medium (20 mL), which contained unreacted substrate, product and mycelium were extracted with ethyl acetate (20 mL). The organic fraction were dried over anhydrous magnesium sulphate and the solvent was evaporated in vacuo and analysed by GC (chiral column).

### 3.9. Biotransformation in the Presence of Deep Eutectic Solvents

In the experiment we either mixed choline chloride (ChCl, 1 mol) with glycerol (Gly, 2 mol) or 2 mol of ChCl with glycerol (1 mol) and glucose (Glc, 1 mol). All DES were maintained at 80 °C for 2 h until a homogeneous liquid was achieved.

The culture of microorganism (20 mL, 12.0 g/L) was centrifuged at 5000 rpm for 3 min. The culture was then re-suspended in 40 mL of sterile phosphate buffer (0.1 M, pH = 7) and then added to appropriate DES (10%, 25%, or 50%). Next, the substrate was added. After a suitable transformation time the medium (20 mL) with unreacted substrate, product and mycelium were extracted with ethyl acetate (20 mL). The organic fraction were dried over anhydrous magnesium sulphate and the solvent was evaporated in vacuo and analysed by GC (chiral column).

## 4. Conclusions

The several biotransformations of α-acetylbutyrolactone **1** were performed using three strains of yeast: *R. glutinis* AM242, *R. marina* AM77 and *R. rubra* C9. The YPG was optimal medium for biotransformation. All strains were capable of converting (±)-α-acetyl-γ-butyrolactone **1** to α’-1′-hydroxyethyl-γ-butyrolactone **2**. Out of the four possible stereoisomers, the most common product was (+)-(3*R*, 1′*S*)-α’-1′-hydroxyethyl-γ-butyrolactone (**2d**). The complete conversion of **1** to **2** occurred within 3 hours for strains *R. marina* AM77 and *R. rubra* C9. The use of resting cells resulted in an increase of reaction time, while *R. marina* AM77 also increased in the enantioselectivity of the process. The addition of 1% glucose to resting cells did not significantly affect the course of biotransformation. Supplementing the YPG medium with different organic solvents had a positive effect on enantioselectivity. Predominantly (with the exception of 20% glycerol) the **2d** stereoisomer was formed only. Unfortunately, the degree of reactivity of **1** decreased considerably. Total conversion of the substrate occurred only in the presence of 5% glycerol. The addition of deep eutectic solvents to yeast resting cells culture negatively affected the course of transformation. There was an increase in biotransformation time and a decrease in stereoselectivity with respect to the pair of *syn* enantiomers. Total reactivity of substrate **1** occurred only when ChCl:Gly was used at 10% and ChCl:Gly:Glc at 25%. In both cases, formation of stereoisomers **2b** and **2d** with high enantiomeric excess was observed.

## Figures and Tables

**Table 1:** The results of biotransformation of α-acetylbutyrolactone **1** by *R. glutinis* AM242 in different media (in % according to GC (chiral column)). YPG: Yeast extract-Peptone-Glucose medium; MCM: Mushroom Complete Medium.

Strain	Medium	Time (h)	Substrate	Stereoisomers of Product			enol (%)
			1 (%)	2a + 2b (*syn*) (%)	2c + 2d (*anti*) (%)	de (%)	
*Rhodotorula glutinis* AM242	A (YPG)	1	83.3	4.6	2.7	26.0	9.4
		2	75.3	8.9	8.0	5.3	7.8
		3	66.3	13.5	14.3	2.9	5.8
		4	52.1	17.8	27.5	21.4	2.6
		5	34.9	22.6	40.1	27.9	2.4
	B (S)	1	85.6	7.8	1.4	69.5	5.2
		2	82.5	10.7	2.5	62.1	3.7
		3	78.5	13.7	4.5	50.5	3.3
		4	70.3	17.3	9.0	31.5	3.4
		5	67.0	15.6	10.0	21.9	7.4
	C (MCM)	1	83.5	5.7	0.5	83.9	10.3
		2	82.3	6.6	1.2	69.2	9.9
		3	78.6	10.4	2.8	57.6	8.2
		4	72.9	11.6	5.1	38.9	10.4
		5	70.2	12.2	6.0	34.1	11.5

**Table 2:** The results of biotransformation of α-acetylbutyrolactone **1** by *R. marina* AM77 in different media (in % according to GC (chiral column)).

Strain	Medium	Time (h)	Substrate	Stereoisomers of Product			enol (%)
			1 (%)	2a + 2b (*syn*) (%)	2c + 2d (*anti*) (%)	de (%)	
*Rhodotorula marina* AM77	A (YPG)	1	77.8	6.8	8.1	8.7	7.2
		2	36.0	15.8	37.8	41.1	10.4
		3	0	21.3	67.6	52.1	11.1
	B (S)	1	86.3	4.0	8.7	37.0	0.9
		2	50.1	11.8	33.1	47.4	5.0
		3	0	19.4	77.2	59.8	3.3
	C (MCM)	1	86.4	4.0	2.8	17.6	6.7
		2	79.6	5.5	7.0	12.0	7.7
		3	48.1	11.2	34.0	50.4	6.7
		4	8.2	14.8	68.3	64.4	8.7
		5	0	12.8	59.8	64.7	27.3

**Table 3:** The results of biotransformation of α-acetylbutyrolactone **1** by *R. rubra* C9 in different media (in % according to GC (chiral column)).

Strain	Medium	Time (h)	Substrate	Stereoisomers of Product			enol (%)
			1 (%)	2a + 2b (*syn*) (%)	2c + 2d (*anti*) (%)	de (%)	
*Rhodotorula rubra* C9	A (YPG)	1	20.9	12.8	62.4	66.0	3.9
		2	0.6	14.2	83.1	70.8	1.8
		3	0	10.1	89.9	79.8	0
	B (S)	1	84.6	7.4	4.0	29.8	4.0
		2	72.6	9.4	12.6	14.5	5.4
		3	43.9	17.5	34.5	32.7	4.1
		4	0	24.8	73.4	49.5	1.8
	C (MCM)	1	85.2	6.6	2.1	51.7	6.0
		2	76.9	10.8	8.3	13.1	4.0
		3	33.4	26.6	32.1	9.4	7.9
		4	0	34.4	60.2	27.3	5.3

**Table 4:** The results of biotransformation of α-acetylbutyrolactone **1** (in % according to GC (chiral column)).

Strain	Time (h)	1 (%)	*syn*		ee (%)	*anti*		ee (%)
			2a (3*S*, 1′*S*) (%)	2b (3*R*, 1′*R*) (%)		2c (3*S*, 1′*R*) (%)	2d (3*R*, 1′*S*) (%)	
*Rhodotorula glutinis* AM242	5	39.3	3.7	17.9	65.7	1.2	38.0	93.9
*Rhodotorula marina* AM77	3	0	2.7	16.4	71.7	0	80.9	100
*Rhodotorula rubra* C9	3	0	1.7	6.8	60.0	0	91.5	100

**Table 5:** The results of biotransformation of α-acetylbutyrolactone **1** in buffer solution (in % according to GC (chiral column)).

Strain	Time (h)	1 (%)	*syn*		ee (%)	*anti*		ee (%)
			2a (3*S*, 1′*S*) (%)	2b (3*R*, 1′*R*) (%)		2c (3*S*, 1′*R*) (%)	2d (3*R*, 1′*S*) (%)	
*Rhodotorula glutinis* AM242	3	90.2	0.3	5.8	90.2	0	3.7	100
	5	87.2	0.3	7.3	92.1	0.2	5.1	92.4
	8	81.4	0.4	9.2	87.3	0.3	8.7	93.5
	24	2.4	3.6	35.7	81.7	0.7	58.6	97.6
	48	0	3.9	36	80.0	0.9	59.2	96.0
*Rhodotorula marina* AM77	3	40.6	0	5.7	100	0	53.7	100
	5	6.2	0	5.5	100	0	88.3	100
	8	0	0	1.6	100	0	98.3	100
*Rhodotorula rubra* C9	3	7.8	4.6	16.8	57.0	0	70.8	100
	5	0	4.6	17.4	58.2	0	78.0	100

**Table 6:** The results of biotransformation of α-acetylbutyrolactone **1** in buffer solution with 1% glucose (in % according to GC (chiral column)).

Strain	Time (h)	1 (%)	*syn*		ee (%)	*anti*		ee (%)
			2a (3*S*, 1′*S*) (%)	2b (3*R*, 1′*R*) (%)		2c (3*S*, 1′*R*) (%)	2d (3*R*, 1′*S*) (%)	
*Rhodotorula glutinis* AM242	3	100	0	0	0	0	0	0
	5	89.6	0.4	5.1	85.5	0	4.9	100
	8	78.5	0.6	7.3	84.4	0	13.6	100
	24	17.6	3.6	32.7	80.2	0.6	45.4	97.4
	48	0	3.4	37.2	83.0	1.1	58.3	96.0
*Rhodotorula marina* AM77	3	57	0.9	6.2	74.6	0	35.9	100
	5	1.3	1.8	9.6	68.0	0	87.3	100
	8	0	0.9	6.7	76.3	0	92.4	100
*Rhodotorula rubra* C9	3	2.9	4.6	10.6	39.0	0	81.9	100
	5	0	3.8	5.4	17.4	0	90.8	100

**Table 7:** The results of biotransformation of α-acetylbutyrolactone **1** by *R. marina* AM77 in mixtures of Medium A and different solvents in different proportions (in % according to GC (chiral column)) after 2 h.

Solvent	% of Solvent	1 (%)	*syn*		ee (%)	*anti*		ee (%)
			2a (3*S*, 1′*S*) (%)	2b (3*R*, 1′*R*) (%)		2c (3*S*, 1′*R*) (%)	2d (3*R*, 1′*S*) (%)	
ethanol	5	52.2	0	0	0	0	47.8	100
	10	87.2	0	0	0	0	12.8	100
	20	99.5	0	0	0	0	0.5	100
glycerol	5	0	0	0	0	0	100	100
	10	7.8	0	0	0	0	92.2	100
	20	80.6	2.8	1.6	27.0	0	15	100
hexane	5	73.9	0	0	0	0	26.1	100
	10	90.8	0	0	0	0	9.2	100
	20	100	0	0	0	0	0	0
isopropanol	5	76.7	0	0	0	0	23.3	100
	10	98.8	0	0	0	0	1.2	100
	20	100	0	0	0	0	0	0

**Table 8:** The results of biotransformation of α-acetylbutyrolactone **1** by *R. marina* AM77 in resting cells culture in the presence of different deep eutectic solvents (DES) (in % according to GC (chiral column)).

DES	Time	1 (%)	*syn*		ee (%)	*anti*		ee (%)
			2a (3*S*, 1′*S*) (%)	2b (3*R*, 1′*R*) (%)		2c (3*S*, 1′*R*) (%)	2d (3*R*, 1′*S*) (%)	
**ChCl:Gly** **10%**	5 h	1.5	0	4.4	100	0	94.1	100
	1 d	0	0	4.7	100	0	95.3	100
**ChCl:Gly** **25%**	5 h	100	0	0	-	0	0	-
	1 d	2.4	2.0	12.8	73.0	0	82.8	100
	2 d	0	2.1	13.3	72.7	0	84.6	100
**ChCl:Gly** **50%**	5 h	100	0	0	-	0	0	-
	1 d	88.8	1.3	6.2	65.3	0	3.7	100
	4 d	31.4	4.8	35.4	76.0	0	27.9	100
	7 d	1.6	5.2	39.8	76.9	0	53.4	100
**ChCl:Gly** **66%**	5 h	100	0	0	-	0	0	-
	1 d	93.7	0.7	4.0	70.2	0	1.6	-
	4 d	76.3	3.2	14.7	64.0	0	5.8	100
	7 d	55.2	8.8	24.6	63.2	0	11.4	100
**ChCl:Gly:Glc 10%**	5 h	4.9	1.6	14.3	80.0	0	79.2	100
	1 d	0	1.4	5.8	61.0	0	92.8	100
**ChCl:Gly:Glc 25%**	5 h	81.2	0	2.5	100	0	16.3	100
	1 d	0	0	13.6	100	0	86.4	100
**ChCl:Gly:Glc 50%**	1 d	83.7	1.7	8.2	66.0	0	6.4	100
	4 d	32.4	6.2	33.8	69.0	0	27.3	100
	7 d	29.5	6.5	31.5	66.0	0	31.8	100
**ChCl:Gly:Glc 66%**	5h	100	0	0	-	0	0	-
	1 d	100	0	0	-	0	0	-
	4 d	92.5	0.6	3.0	67.0	0	3.2	100
	7 d	87.5	2.1	6.0	65.0	0	4.4	100

ChCl:Gly—choline chloride and glycerol 1:2; ChCl:Gly:Glc—choline chloride, glycerol, and glucose 2:1:1. d: days.

## Supplementary Materials

Supplementary materials can be found at http://www.mdpi.com/1422-0067/19/7/2106/s1.
