# Supplementary material for: Biotransformation of α-Acetylbutyrolactone in Rhodotorula Strains
Source: Int J Mol Sci. 2018 Jul 20;19(7):2106. doi: 10.3390/ijms19072106 (PMC6073705; doi:10.3390/ijms19072106)
Supplement: Supplementary file 1 [file ijms-19-02106-s001.pdf]

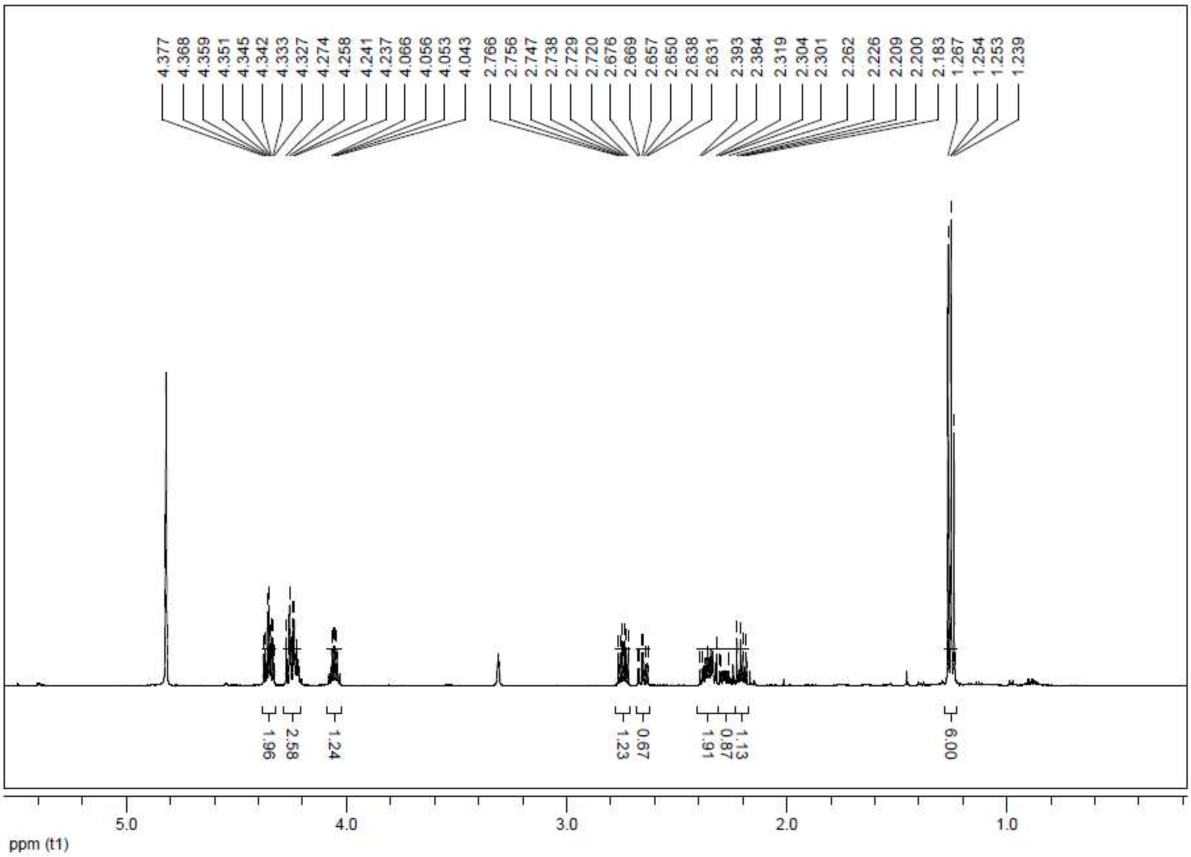

Figure S1. ^{1}H NMR (500 MHz, CD_3OD) spectrum of α'-1'-hydroxyethyl-γ-butyrolactone **2.**

S1 Preparation of (3*R*, 1′*R*)-α'-1'-hydroxyethyl-γ-butyrolactone 2

Yarrowia lipolytica P26A was cultivated at 25 °C in two Erlenmayer flasks (300 mL) containing 50 mL of YPG medium (glucose 20 g/L, peptone 20 g/L, yeast extract 10 g/L). After 4 days of culture growth, 50 µL of substrate (0.060 g, 7.8 mmol) was added to shaken cultures. After one day, the medium contained unreacted substrate, product and mycelium was extracted with ethyl acetate (100 mL). The organic fraction was dried over anhydrous magnesium sulphate, the solvent was evaporated *in vacuo* and analysed by GC (chiral column). Product of biotransformation was purified by means of column chromatography (Kieselgel 60, 230–400 mesh; hexane:diethyl ether 1:1). Next, the optical rotation was determined. The obtained value of optical rotation ($[a]_{20}^{D}$= −20.4 (c = 0.9; $CHCl_3$) was compared with literature data. [13] The configuration of biotransformation product was determined as (3*R*, 1′*R*).
